# Ursodeoxycholic acid enriches intestinal bile salt hydrolase-expressing *Bacteroidetes* in cholestatic pregnancy

**DOI:** 10.1038/s41598-020-60821-w

**Published:** 2020-03-03

**Authors:** Caroline Ovadia, Alvaro Perdones-Montero, Hei Man Fan, Benjamin H. Mullish, Julie A. K. McDonald, Georgia Papacleovoulou, Annika Wahlström, Marcus Ståhlman, Anastasia Tsakmaki, Louise C. D. Clarke, Alexandros Sklavounos, Peter H. Dixon, Gavin A. Bewick, Julian R. F. Walters, Hanns-Ulrich Marschall, Julian R. Marchesi, Catherine Williamson

**Affiliations:** 1 Maternal and Fetal Disease Group, Department of Women and Children’s Health, King’s College London, London, SE1 1UL UK; 2 Section of Biomolecular Medicine, Division of Computational & Systems Medicine, Department of Surgery & Cancer, Faculty of Medicine, Imperial College London, London, SW7 2AZ UK; 3 Centre for Clinical Microbiome Research and The Division of Integrative Systems Medicine and Digestive Disease, Department of Surgery and Cancer, Imperial College London, London, W2 1NY UK; 4 Sahlgrenska Academy, Institute of Medicine, Department of Molecular and Clinical Medicine, University of Gothenburg, Gothenburg, 41345 Sweden; 5 Diabetes Research Group, School of Life Course Sciences, King’s College London, London, SE1 1UL UK; 6 Division of Digestive Diseases, Hammersmith Hospital, Imperial College London, London, W12 0HS UK; 7 Cardiff School of Biosciences, Cardiff University, Cardiff, CF10 3XQ UK

**Keywords:** Microbiome, Endocrine reproductive disorders

## Abstract

Ursodeoxycholic acid (UDCA) treatment can reduce itch and lower endogenous serum bile acids in intrahepatic cholestasis of pregnancy (ICP). We sought to determine how it could influence the gut environment in ICP to alter enterohepatic signalling. The gut microbiota and bile acid content were determined in faeces from 35 pregnant women (14 with uncomplicated pregnancies and 21 with ICP, 17 receiving UDCA). Faecal bile salt hydrolase activity was measured using a precipitation assay. Serum fibroblast growth factor 19 (FGF19) and 7α-hydroxy-4-cholesten-3-one (C4) concentrations were measured following a standardised diet for 21 hours. Women with a high ratio of *Bacteroidetes* to *Firmicutes* were more likely to be treated with UDCA (Fisher’s exact test p = 0.0178) than those with a lower ratio. Bile salt hydrolase activity was reduced in women with low *Bacteroidetes*:*Firmicutes*. Women taking UDCA had higher faecal lithocholic acid (p < 0.0001), with more unconjugated bile acids than women with untreated ICP or uncomplicated pregnancy. UDCA-treatment increased serum FGF19, and reduced C4 (reflecting lower bile acid synthesis). During ICP, UDCA treatment can be associated with enrichment of the gut microbiota with *Bacteroidetes*. These demonstrate high bile salt hydrolase activity, which deconjugates bile acids enabling secondary modification to FXR agonists, enhancing enterohepatic feedback via FGF19.

## Introduction

The serum and faecal bile acid composition is intimately related to biotransformation of bile acids by intestinal bacteria, and their subsequent enterohepatic circulation. Deconjugation of primary bile acids by bacterial bile salt hydrolase (BSH) enables unconjugated bile acids to be modified to secondary bile acids. Bile acids act as signalling molecules for many different end organs (e.g. liver, pancreas, adipose tissue, inflammatory cells), with individual bile acid species of differing ligand potency for different receptors (e.g. farnesoid X receptor (FXR), Takeda G-protein-coupled receptor 5 (TGR5))^1–3^.

Intrahepatic cholestasis of pregnancy (ICP) is predominantly a liver disorder specific to pregnancy, defined by pruritus and elevated serum bile acids beyond the normal asymptomatic hypercholanaemia of pregnancy. Fetal adverse outcomes are related to the extent of elevation of serum concentrations of total bile acids^4,5^. Women with ICP have increased rates of impaired glucose tolerance, gestational diabetes mellitus, and dyslipidaemia^6,7^. The drug ursodeoxycholic acid (UDCA) improves itch severity and alters the composition of the serum bile acid pool in ICP^8,9^. Previous studies have suggested that UDCA may be of additional benefit for women with ICP, for example by normalising the ICP-related fall in glucagon-like peptide 1 (GLP1) release following a meal^6^. Indeed, murine studies have demonstrated that UDCA treatment can lower blood glucose concentrations in mice fed a high-fat diet^10^.

A number of studies have established that the gut microbiota changes during pregnancy, and this can be associated with the gestational metabolic alterations observed in late pregnancy^11–13^. We hypothesised that the metabolic improvements associated with UDCA treatment of ICP are contributed to by the beneficial effects of an altered intestinal microbiota, providing enhanced enterohepatic feedback. Human studies of the intestinal microbiota are complicated by inter-individual differences in diet, environment and genetics. Furthermore, the composition of the intestinal content must be inferred from faecal samples, particularly during pregnancy, when endoscopy for research is relatively contra-indicated. We therefore used a murine model to further interrogate the effects observed in humans. Cholic acid (CA) dietary supplementation has previously been demonstrated to result in serum bile acid concentrations comparable to those observed in ICP^14^; we used this model in combination with UDCA dietary supplementation to assess effects on the caecal gut microbiota to support our human results.

## Results

### Human intestinal microbiota in normal and UDCA-treated cholestatic pregnancies

To determine the effect of UDCA treatment on the composition of the gut microbiota, metataxonomics was performed on faecal samples from fourteen women with normal pregnancies, four women with untreated ICP, and seventeen women with UDCA-treated ICP (Supplementary Table S1).

**Figure 1:**
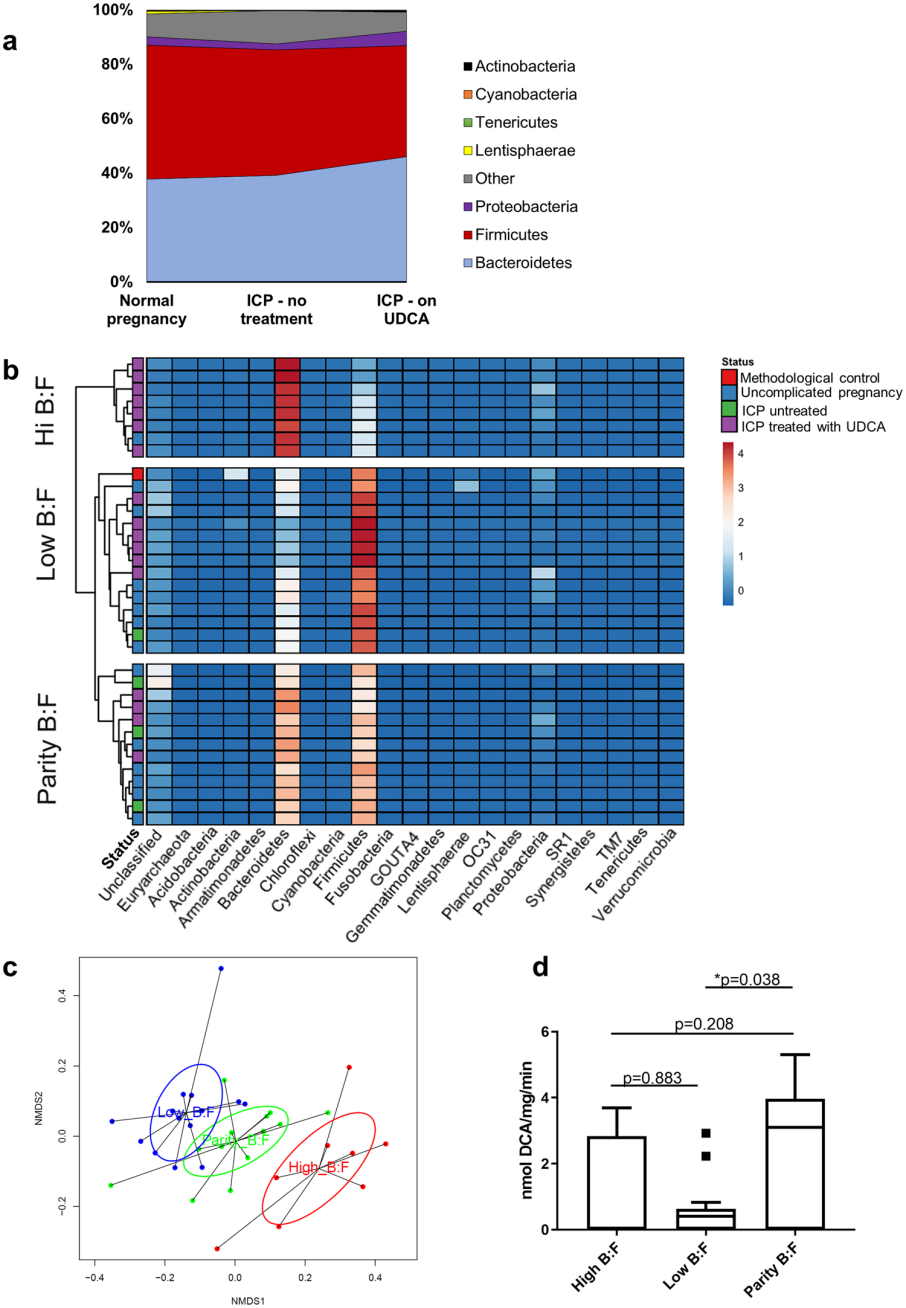
The faecal microbiota profiles of cholestatic and uncomplicated pregnancy cluster according to the ratio of *Bacteroidetes* to *Firmicutes*. **(a)** Distribution of microbes at phylum level in women with normal pregnancies (n = 14), women with intrahepatic cholestasis of pregnancy (ICP) treated with ursodeoxycholic acid (UDCA, n = 17), and women with untreated ICP (n = 4). **(b)** Heat map demonstrating unsupervised clustering of faecal samples by microbiota profiles determined from 16 S rRNA gene sequencing, according to *B*:*F* (the ratio of *Bacteroidetes* to *Firmicutes*). Each row refers to faecal samples from individual women as per (a), uncomplicated pregnancy: blue status; ICP treated with UDCA: purple status; untreated ICP: green status; red status shows methodological control. Box colours show relative bacterial abundance, dark blue reflecting minimal sequences present in samples – red showing high sequence levels in samples. **(c)** NMDS analysis of 16 S rRNA gene sequences from human faecal samples as per (a), demonstrating clustering according to ratio of *Bacteroidetes* to *Firmicutes* (*B*:*F*). Red: high *B*:*F*, blue: low *B*:*F*, green: parity of *B*:*F*. **(d)** Bile salt hydrolase activity of human faecal samples as per (a), according to ratio of *Bacteroidetes* to *Firmicutes* (*B*:*F*), determined by nmol of deoxycholic acid (DCA) production per mg of protein per minute. Tukey box-plots show median, IQR and whiskers at 1.5 IQR. Significance determined by one-way ANOVA with Tukey *post hoc* comparison, ANOVA (F(2,32  =  3.55), p = 0.040); *p = 0.038.

There was an overall increase in the relative abundance of *Bacteroidetes* compared with *Firmicutes*, the two most populous phyla in the colonic microbiota, in the women treated with UDCA (Fig. 1a, Supplementary Fig. S1). Unsupervised hierarchical clustering revealed that the faecal samples clustered into three groups according to the ratio of *Bacteroidetes* to *Firmicutes* (Fig. 1b,c), and this clustering continued to order level, revealing the same groups with the ratio of *Bacteroidales* to *Clostridiales* (Supplementary Fig. S2). Women with a high *Bacteroidetes* to *Firmicutes* ratio were more likely to be treated with UDCA than women with lower ratios (p = 0.0178, Fisher’s exact test compared with both low and parity of *Bacteroidetes*:*Firmicutes*, p = 0.0412, Freeman Halton extension of Fisher’s exact test compared between each cluster). For the women treated with UDCA, those with a high ratio of *Bacteroidetes* to *Firmicutes* received a greater total dose of UDCA prior to the sample being collected (p = 0.004) than those with parity or a low ratio; there was no other difference between the groups (Table 1).

**Table 1:** Clinical features of women treated with UDCA based upon the ratio of *Bacteriodetes* to *Firmicutes*.

	High *B:F* (n = 7) Median (IQR)	Low or Parity *B:F* (n = 10) Median (IQR)	Comparison
Maternal age (years)	36 (34 to 40)	35 (29 to 38)	ns
Gestation itch commenced (week^+day^)	28^+3^ (21^+0^ to 29^+0^)	32^+4^ (29^+0^ to 34^+4^)	ns
Gestation of sample (week^+day^)	35^+5^ (30^+5^ to 36^+5^)	36^+5^ (34^+4^ to 37^+1^)	ns
UDCA total dose prior to sample (g)*	76 (15 to 92)	10 (1 to 19)	p = 0.004
Peak bile acid concentration pre-sample (µmol/L)	65 (41 to 214)	40 (26 to 75)	ns
Bile acid concentration at time of sample (µmol/L)	46 (21 to 136)	29 (19 to 50)	ns
Peak bile acid concentration throughout pregnancy (µmol/L)	78 (64 to 254)	60 (29 to 109)	ns

*B*: *Bacteroidetes*; *F*: *Firmicutes*; n: number; IQR: interquartile range; ns: not significant. *Total UDCA dose assuming 100% compliance, calculated using prescribed dose(s) and duration. Comparisons using two-tailed student’s t-tests, p < 0.05 defined as threshold of significance.

We have previously demonstrated in mice that caecal *Bacteroidetes-*encoded BSH capacity increased in pregnancy and in a model of CA dietary supplementation^13^. An assay of BSH activity was therefore performed, which demonstrated that faecal samples with lower *Bacteroidetes* than *Firmicutes* indeed did have reduced enzymatic activity (p = 0.0379) (Fig. 1d).

### Faecal bile acid profile in women with cholestatic and normal pregnancies, demonstrating the effect of UDCA treatment

**Figure 2:**
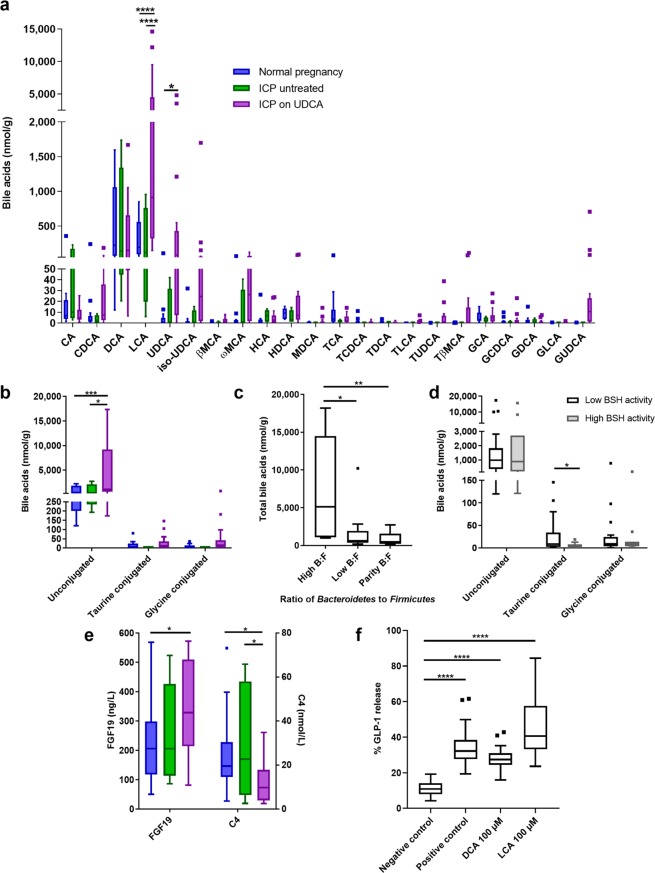
Women treated with ursodeoxycholic acid for cholestatic pregnancy have altered faecal bile acids and enhanced enterohepatic feedback. **(a)** Faecal bile acids from women in the third trimester of uncomplicated pregnancy (blue boxes, n = 14), untreated ICP (green boxes, n = 4), and ICP treated UDCA (purple boxes, n = 17). Groups were compared with 2-way ANOVA with Tukey’s multiple comparisons test; ****adjusted p < 0.0001, *adjusted p = 0.0294. CA: cholic acid, CDCA: chenodeoxycholic acid, DCA: deoxycholic acid, LCA: lithocholic acid, MCA: muricholic acid, HCA: hyocholic acid, HDCA: hyodeoxycholic acid, MDCA: murideoxycholic acid, TCA: taurocholic acid, TCDCA: taurochenodeoxycholic acid, TDCA: taurodeoxycholic acid, TLCA: taurolithocholic acid, TUDCA: tauroursodeoxycholic acid, TβMCA: taurobetamuricholic acid, GCA: glycocholic acid, GCDCA: glycochenodeoxycholic acid, GDCA: glycodeoxycholic acid, GLCA: glycolithocholic acid, GUDCA: glycoursodeoxycholic acid. (**b**) Faecal bile acids by conjugation, from samples as per (**a**). Groups were compared with 2-way ANOVA with Tukey’s multiple comparisons test; ***adjusted p = 0.0003, *adjusted p = 0.0277. (**c**) Faecal bile acid levels according to ratio of *Bacteroidetes* to *Firmicutes* (B:F), determined by unsupervised clustering, from samples as per (**a**). Groups compared with Kruskal-Wallis test with Dunn’s multiple comparisons test; **adjusted p = 0.0086, *adjusted p = 0.0470. (**d**) Faecal bile acid levels by conjugation according to bile salt hydrolase (BSH) activity. Low BSH activity (white boxes): 0.00–0.83 nmol DCA/mg/min (n = 24), high BSH activity (grey boxes): 2.23–5.31 nmol DCA/mg/min (n = 11). Groups compared with Mann-Whitney tests, *p = 0.0106. (**e**) Serum fibroblast growth factor 19 (FGF19) and 7α-hydroxy-4-cholesten-3-one (C4) concentrations from women in the third trimester of uncomplicated pregnancy (blue boxes, n = 24), untreated ICP (green boxes, n = 10) and UDCA-treated ICP (purple boxes, n = 10). Samples were taken at 15:00, following a standardized diet for 21 hours. Groups compared with multiple t tests, and Holm-Sidak correction for multiple testing. For FGF19: *p = 0.0302, for C4: normal vs ICP on UDCA *p = 0.0296, ICP untreated vs ICP on UDCA *p = 0.0335. (f) Percentage of glucagon-like peptide one (GLP1) released from murine colonic tissue on exposure to the bile acids LCA and DCA. Negative control – buffer only, positive control: 10 µM 3-isobutyl-1-methylxanthine (IBMX) with 10 µM forskolin (n = 4, with 7–8 replicates per experiment). Boxes show median and interquartile range (IQR), with whiskers at 1.5 IQR.

Faecal samples were subsequently assayed to determine bile acid composition. In UDCA-treated women with ICP, UDCA and its metabolite, lithocholic acid (LCA), predominated (Fig. 2a). This group also had significantly higher proportions of unconjugated bile acids than those with normal pregnancies (Fig. 2b). Faecal samples with a higher ratio of *Bacteroidetes*:*Firmicutes* had significantly more bile acids per gram than those with low or parity of *Bacteroidetes*:*Firmicutes* (Fig. 2c); this was true for both unconjugated and conjugated bile acids. In turn, high BSH activity was associated with reduced taurine-conjugated bile acids (Fig. 2d).

We have previously demonstrated the effect of UDCA treatment on individual serum bile acids^15^, with UDCA comprising approximately 60% (42.8–69.0%, median (IQR)) of the bile acid pool in treated, and 0.3% (0.0–0.9%) in untreated women. To determine the relative effect on classical and alternative pathways of bile acid synthesis, we used this dataset to compare the ratio of CA to chenodeoxycholic acid (CDCA) following treatment (Supplementary Fig. S3). The proportion of CA reduced compared to CDCA for women who had taken at least 14 g UDCA (p = 0.04), with 83% (15/18) with lower CA:CDCA than prior to treatment (Supplementary Table S2).

Since bile acids have different potencies with respect to FXR activation, we assessed the impact of ICP on intestinal FXR signalling by measuring the serum concentration of FGF19 in a separate cohort of women with ICP and normal pregnancies given a standardised diet over 24 hours. Treatment with UDCA significantly increased peak circulating FGF19 (Fig. 2e) with a corresponding reduction in serum 7α-hydroxy-4-cholesten-3-one (C4) concentration. Similarly, the effects of the secondary metabolites of UDCA on intestinal release of GLP1 were determined using primary murine colonic cultures. Incubation with LCA and deoxycholic acid (DCA) resulted in release of GLP1 from intestinal crypts that was not seen in control incubations (Fig. 2f).

### Murine model of dietary hypercholanaemia, to determine the effects of UDCA treatment

**Figure 3:**
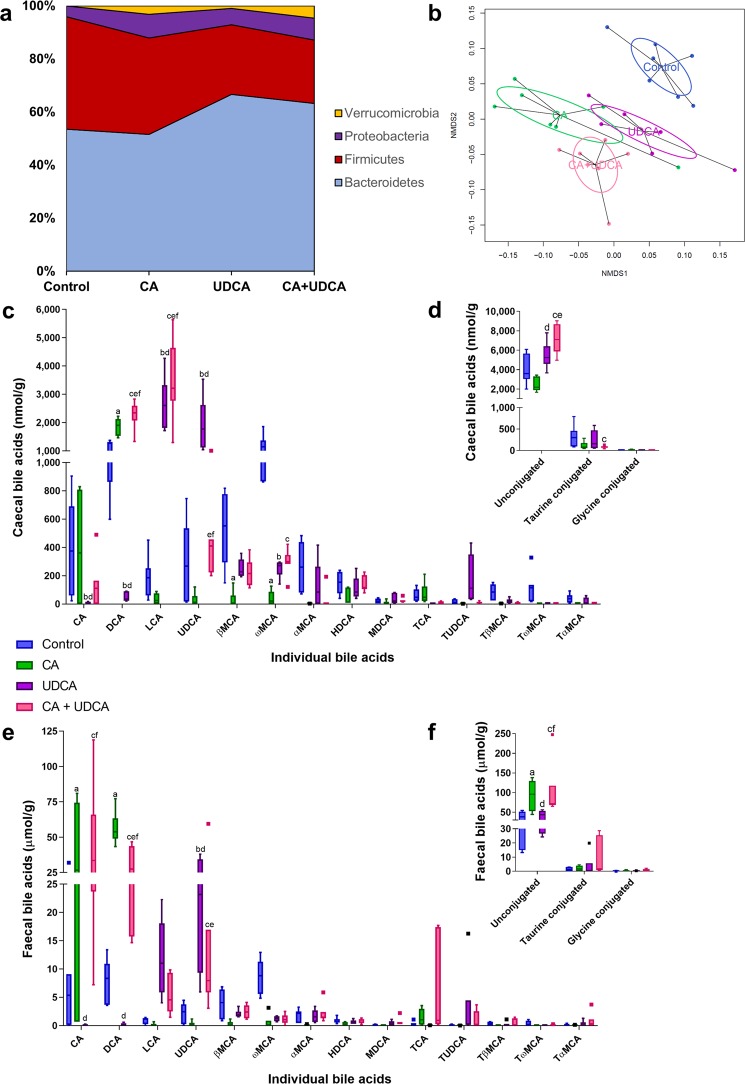
Ursodeoxycholic acid dietary supplementation for pregnant mice increases caecal *Bacteroidetes* and unconjugated bile acids. (**a)** Distribution of microbes at phylum level in day 18 pregnant mice given a normal chow (control) diet (n = 7), 0.5% cholic acid (CA) supplemented diet (n = 7), 0.5% ursodeoxycholic acid (UDCA) supplemented diet (n = 6) and 0.5% CA + 0.5% UDCA supplemented diet (n = 7). **(b)** NMDS plot demonstrating distribution of gut microbiota according to diet, for mice as per (a). **(c)** Caecal bile acids from pregnant mice as per (a). Control: blue boxes, 0.5% CA diet: green boxes, 0.5% UDCA diet: purple boxes, 0.5% CA + 0.5% UDCA diet: pink boxes. DCA: deoxycholic acid, LCA: lithocholic acid, MCA: muricholic acid, HDCA: hyodeoxycholic acid, MDCA: murideoxycholic acid, TCA: taurocholic acid, TUDCA: tauroursodeoxycholic acid, TβMCA: tauro-betamuricholic acid, TωMCA: tauro-omegamuricholic acid, TαMCA: tauro-alphamuricholic acid. **(d)** Caecal bile acids by conjugation, from pregnant mice as per (a). Control: blue boxes, 0.5% CA diet: green boxes, 0.5% UDCA diet: purple boxes, 0.5% CA + 0.5% UDCA diet: pink boxes. **(e)** Faecal bile acids from pregnant mice as per (a). Control: blue boxes, 0.5% CA diet: green boxes, 0.5% UDCA diet: purple boxes, 0.5% CA + 0.5% UDCA diet: pink boxes. **(f)** Faecal bile acids by conjugation, from pregnant mice as per (a). Control: blue boxes, 0.5% CA diet: green boxes, 0.5% UDCA diet: purple boxes, 0.5% CA + 0.5% UDCA diet: pink boxes. Groups were compared with 2-way ANOVA with Tukey’s multiple comparisons test; adjusted p values where p < 0.05: a control vs CA; b control vs UDCA; c control vs CA+UDCA, d CA vs UDCA; e CA vs CA+UDCA; f UDCA vs CA+UDCA. Boxes show median and interquartile range (IQR), with whiskers at 1.5 IQR.

To support these findings, we performed metataxonomics on the caecal content of mice fed a normal chow control diet, and diets supplemented with 0.5% CA, 0.5% UDCA and 0.5% CA plus 0.5% UDCA immediately prior to and during pregnancy. Dietary supplementation with bile acids significantly altered the composition of the gut microbiota, with mice clustering according to their diet groups (Fig. 3a,b, Supplementary Table S3, Supplementary Fig. S4). Bacterial richness decreased with supplementation with any bile acid, and diversity reduced when UDCA was included in the diet only (Supplementary Fig. S5). Increases in *Proteobacteria*, in particular, were seen with CA supplementation, whilst UDCA diets were associated with increased *Bacteroidetes*, similar to the changes observed in human faeces (Fig. 3a). Caecal bile acid levels reflected the dietary bile acid load, in particular with higher secondary bile acids produced by bacterial modification of the supplemented bile acids (DCA from CA, LCA from UDCA) (Fig. 3c). Conversely, ω-muricholic acid (ωMCA) is produced by bacterial modification of α- and βMCA, which are produced by *de novo* hepatic synthesis; this was significantly lower for mice from all groups supplemented with dietary bile acids. Further, UDCA-supplemented diets resulted in higher unconjugated bile acids than control or CA supplementation alone (Fig. 3d).

Similar changes to the caecal findings were seen in the bile acid content of the corresponding murine faeces (Fig. 3e). Notable exceptions were that CA supplementation resulted in significantly higher CA levels in the faeces of supplemented mice than controls, which was not seen in the caecum; and the highly significant elevations of LCA with UDCA treatment found in the caecum were not present in the faeces. In contrast to the caecal results, CA supplementation resulted in higher levels of unconjugated bile acid than control or UDCA-fed mice (Fig. 3f).

## Discussion

This study has identified that UDCA-treatment in cholestatic pregnancy can alter the composition of gut microbiota, increasing the proportion of *Bacteroidetes* with associated increased BSH activity. This change results in an altered intestinal bile acid environment, with more unconjugated bile acids available for enhanced secondary bile acid production, and increased FGF19-mediated enterohepatic feedback. Lower C4 concentrations were consistent with increased negative feedback on *de novo* hepatic bile acid synthesis through fibroblast growth factor receptor 4/ beta-klotho hepatic signalling. This finding is consistent with reports of reduced serum concentrations of the endogenous primary bile acids in UDCA-treated women^15^.

Observational studies of human disease are subject to many confounders, which are particularly difficult to control for in pregnancy. A limitation of this study is the interindividual variability present for the relatively small number of women participating, for whom other clear confounders have been excluded. To address this, we used a murine model of cholestatic pregnancy supplemented with oral UDCA, and examined how the caecal intestinal microenvironment was affected by UDCA treatment. At the phylum-level, UDCA-supplementation of mice resulted in similar alterations to those seen for women with ICP treated with UDCA, notably a higher proportion of *Bacteroidetes* with a correspondingly lower proportion of *Firmicutes*. Previous studies have demonstrated that dietary cholic acid-associated enrichment of the microbiota with *Bacteroidetes* was associated with increased *bsh* gene read counts, and these originated from this group of bacteria^13^. Our findings are consistent with this observation following UDCA treatment, as this resulted in higher proportions of unconjugated bile acids in the caecum compared to control mice, and no corresponding rise in taurine-conjugated bile acids, which would be expected if this resulted entirely from the additional dietary load.

Ursodeoxycholic acid may reduce the non-UDCA total serum bile acids for women with ICP^8,9,15^. This mechanism of action has been attributed to increased hepatic bile acid secretion, with enhanced choleresis secondary to vesicular exocytosis (reviewed by Beuers *et al*.)^16^. Herein, we provide a complementary explanation: by enhancing enterohepatic feedback via FGF19, UDCA can reduce hepatic bile acid synthesis *de novo*.

FGF19 is produced upon bile acid ligand binding to FXR, although UDCA itself is not a strong FXR agonist in intestinal cells^17^. UDCA is modified by intestinal bacteria to produce alternative secondary bile acids; 7β-dehydroxylation results in the formation of LCA, which explains its significantly higher levels in the faeces of UDCA-treated women with ICP than other pregnant groups; but LCA also causes induction of intestinal FXR to produce FGF19^18^. UDCA can also be converted by bacterial 7α- and 7β-hydroxysteroid dehydrogenase into the potent intestinal FXR agonist CDCA^18,19^. Although it did not reach statistical significance after adjustment for multiple comparisons, an increase in CDCA was observed in the faeces of UDCA-treated women; it is likely that levels of CDCA are higher in the terminal ileum than the faeces since it is efficiently uptaken by passive diffusion, via ASBT when conjugated, or biotransformed to LCA (reviewed by Crosignani *et al*.)^20^. Thus, CDCA agonism may explain the increased FGF19 levels seen in UDCA-treated women. Mechanisms to safely obtain terminal ileal content samples in pregnant women would be required to support this conclusion.

We confirmed that an increase in the CDCA:CA ratio occurs in the serum of women with ICP during UDCA treatment^15,21^. We conclude that hepatic bile acid synthesis via the classical pathway to produce CA is more affected by UDCA treatment than intra- and extra-hepatic synthesis of CDCA via the alternative pathway, secondary to the hepatic action of FGF19. This is consistent with the effect of FGF19 analogue administration, where the bile acid profile in mice reduces CA in favour of MCA synthesis^22^. In the murine model, treatment with UDCA alone or in combination with CA did not reduce βMCA. However, UDCA alone did reduce CA, demonstrating that its use could alter the balance of classical/alternative bile acid synthesis pathways in the liver, similar to humans.

The difference in our findings between enhancement of FGF19 release compared with the reductions seen in morbidly obese individuals treated with UDCA^23^ may reflect the different underlying states of study individuals. Distal ileal FXR expression has been correlated with body mass index, with obese individuals having 3-fold higher FGF19 mRNA measured^24^. Notably, ICP is not associated with maternal obesity^25^. Furthermore, the effects of pregnancy and obesity on the composition of the gut microbiota differ considerably: pregnancy is associated with an increase in bacterial diversity and higher *Bacteroidetes*^13^ whilst a number of studies of obesity report reduced diversity with lower *Bacteroidetes*^26–29^. Our findings are consistent with those following faecal microbiota transplant for the treatment of *Clostridioides difficile*, which results in enhanced FGF19 enterohepatic feedback secondary to restoration of intestinal bacteria, in particular those encoding BSH activity^30^. Additionally, ileal apical sodium-dependent bile acid transporter (ASBT) protein levels fall in pregnancy^13^, for which conjugated bile acids are the preferred substrate for bile acid uptake by the enterocyte. Thus, passive diffusion of unconjugated bile acids is likely of greater relevance in pregnancy to affect subtrate availability for intestinal FXR signalling, which is dose dependent^31^; this is consistent with findings that unconjugated bile acids (CDCA and DCA) increase FGF19 gene expression in two intestinal cell lines (Caco-2 and T84)^32^.

Additionally, this study suggests a mechanism by which treatment with UDCA might improve GLP1 release (and thus impaired glucose tolerance) in ICP. The marked elevation of LCA in the faeces of treated women is likely to provide a local agonist to intestinal enteroendocrine L cells, which we confirmed in explants triggers GLP1 release, likely by signalling via TGR5. This result is consistent with a recent meta-analysis of seven studies (626 participants) assessing glycaemic markers in patients treated with UDCA, which found reductions in fasting blood glucose, glycosylated haemoglobin and insulin levels compared to control patients, in studies in non-alcoholic steatohepatitis, NAFLD and type 2 diabetes mellitus^33^.

This study supports the conclusion that the secondary modifications of UDCA by bacteria to metabolically-active bile acids are important in delivering the intestinally-derived benefits of UDCA treatment in ICP. Although unconjugated UDCA is delivered to the intestines from the ingested medication for treated women, the efficiency of the enterohepatic circulation and subsequent conjugation in the liver enable subsequent biliary secretion of conjugated UDCA. Modification of this to LCA or CDCA requires cleavage from the conjugated bile acid by bacterial BSH . Hence, we conclude that an intestinal environment favourable to bile salt hydrolase-producing bacteria is likely to enhance enterohepatic feedback. Women with a high ratio of *Bacteroidetes*:*Firmicutes* were more likely to be taking UDCA (7/8), however others taking UDCA had a low ratio (6/14), and correspondingly reduced BSH activity in the faeces. This biological variation may explain the differing clinical responses to UDCA that we have observed for women with ICP^34^, and provide an additional treatment target (enriching the intestinal microbiota with BSH-producing bacteria) to provide in combination with UDCA for the treatment of ICP. Identification of women with a high *Bacteroidetes*:*Firmicutes* signature prior to treatment may predict better response to treatment with UDCA, and future studies could use this as a predictive tool to treatment response. Alternatively, the observation that women with a high ratio of *Bacteroidetes*:*Firmicutes* had taken significantly more UDCA prior to sample donation than those women with a lower ratio suggests that there is a dose-response to UDCA treatment that may affect the composition of the gut microbiota. The lower quartile of UDCA exposure for women with high *Bacteroidetes*:*Firmicutes* was more than 2 g/day for one week, providing the first evidence for minimal effective dosing for UDCA in ICP.

## Methods

### Human studies

The study was approved by the ethics committee of Hammersmith Hospitals NHS Trust, London (08/H0707/21 and 11/LO/0396), and performed according to the principles of the 1975 Declaration of Helsinki. Written informed consent was obtained from all participants prior to inclusion in the study. Women were opportunistically recruited in the third trimester of pregnancy to donate faecal samples; these were obtained from 21 women with ICP and 14 women with uncomplicated pregnancies. ICP was confirmed by demonstration of serum bile acids > 10 µmol/L in association with pruritus, with no additional identifiable cause for their liver dysfunction. Women were restricted to those with spontaneously conceived third trimester singleton pregnancies, who had not taken antibiotics for the duration of the pregnancy, and did not report any other pregnancy complications. Faecal samples were frozen at −80 °C within 24 hours of sample collection, and sections from the same sample used for metataxonomics, bile acid measurement and BSH activity assays.

Serum samples were obtained from women following a standardised diet from 18:00 the preceding day. Serum FGF19 and C4 levels were analysed as previously described^13^ from serum samples obtained at 15:00 (correspondent with peak FGF19) for 24 women with uncomplicated pregnancies, and 20 women with ICP (10 untreated, and 10 treated with UDCA). The measurement of individual serum bile acids was previously described in a study of the effect of UDCA treatment on serum bile acid profile^15^; we used these data to calculate the ratio of CA:CDCA for new comparison.

### Murine studies

The experiments were conducted according to the UK Animals (Scientific Procedures) Act of 1986, with approval of the King’s College London Animal Studies Committee. Age-matched female C57BL/6 mice (Harlan Laboratories, Bicester, UK) were housed in the same room, with a 12-hour light cycle, with 3 mice per cage according to dietary group. Mice were fed, *ad libitum* a normal chow (RM3, Special Diets Services, Essex, UK) control diet, or an RM3 diet supplemented with 0.5% CA, 0.5% UDCA, or 0.5% CA plus 0.5% UDCA (LBS Biotechnology, Horley, UK, n = 6–7 per group), and after one week were mated. Mice were sacrificed at day 18 of pregnancy, at which time caeca and faeces were harvested, snap frozen on dry ice and stored at −80 °C.

Primary colonic culture secretion studies were performed as previously described^35^. In brief, male C57BL/6 mice fed a control diet were sacrificed at 10–12 weeks of age, and colons were harvested. 1mm^2^ squares of cleaned colon were digested with collagenase from *Clostridium histolyticum* (Sigma-Aldrich, St Louis, US) in Dulbecco’s-modified Eagle medium (DMEM) (Sigma-Aldrich, St Louis, US), and cultured overnight on 1% Matrigel-coated plates (Corning, New York, US) and DMEM with 10% fetal calf serum and 1% penicillin and streptomycin(Sigma-Aldrich, St Louis, US). Cultures were treated with 100 µM LCA or 100 µM DCA, and 10 µM 3-isobutyl-1-methylxanthine (IBMX)/forskolin used as a positive control, for 2 hours. GLP1 concentrations were measured by ELISA (Millipore Sigma, Burlington, US) for the supernatant and lysed cells, and GLP1 release calculated as a percentage of total levels.

### Metataxonomic sequencing

Murine caecal content was separated from overlying intestine whilst frozen with macroscopic dissection. Human faecal aliquots (200 mg) and murine caecal content were lysed using the Qiagen Tissuelyser II bead beater (25 Hz for 20 minutes), with DNA extracted using the QiaAMP Fast DNA Stool Mini Kit (Qiagen, Venlo, Netherlands), according to manufacturer’s instructions.

16S rRNA gene sequencing was performed with the Illumina MiSeq platform (Illumina Inc., Saffron Waldon, UK). Human faecal 16S rRNA gene sequencing using V1-V3 primers was performed by Research and Testing Laboratories, Texas, as per published protocols^36^, whilst murine caecal 16S rRNA gene sequencing was performed using V1-V2 primers in house^37^. Murine caecal sample libraries were cleaned and normalised using the SequalPrep Normalization Plate Kit (Life Technologies, Paisley, UK). Sample library quantification was performed with the NEBNext Library Quant Kit for Illumina (New England Biolabs, Hitchin, UK), and 300 bp paired-end sequencing performed using the MiSeq Reagent Kit v3 (Illumina). Data were analysed using Mothur software^38^, with nucleic acid sequences aligned using the SILVA database^39^ and classified using the ribosomal data project (RDP) database reference sequence files according to Wang *et al*.^40^. Statistical analyses were performed in R, using the Vegan package to perform non-metric multidimensional scaling (NMDS) and permutational multivariate analysis of variation (PERMANOVA). The Statistical Analysis of Metagenomic Profiles (STAMP) software was used to compare groups at taxonomic levels, using the Kruskal-Wallis H-test with Tukey-Kramer post hoc testing and correction for multiple testing with Benjamini-Hochberg FDR. Alpha diversity (Shannon diversity index) and richness (total number of bacterial taxa observed) calculated in Mothur were compared using SPSS version 23 (IBM, New York, USA).

### Bile salt hydrolase activity assay

Faecal water was prepared and total faecal protein quantified similarly to a method previously-described^41^, but with the addition of bacterial and mammalian protease inhibitor cocktails (G Biosciences, Uttar Pradesh, India), as well as Dithiothreitol to 1 mM final concentration (Roche, Basel, Switzerland) (to minimise enzyme oxidation^42^).

The BSH assay has been described previously^37^. In brief, BSH activity was determined by measuring insoluble DCA precipitated (determined by absorbance at 600 nm (A_600_)) following incubation of 500 µg of faecal protein with taurodeoxycholic acid (Sigma-Aldrich, St Louis, US). Samples were compared with a standard curve of known DCA concentrations and measured in triplicate.

### Bile acid quantification

Faecal and caecal samples were homogenized in methanol (containing internal standards) with ceramic beads using the Qiagen Tissuelyser II as previously described^43^. Following centrifugation, 20 µL supernatant was diluted with 980 µL MeOH:H2O 1:1. Bile acids were separated and detected using ultra-performance liquid chromatography coupled to mass spectrometry, as previously reported^43^. Quantification was made using an external standard curve.

### Statistical analyses

Where not otherwise indicated in the methods, results were compared using GraphPad Prism (version 7.02) using Fisher’s exact test, analysis of variation (ANOVA) and Tukey’s multiple comparisons test (accounting for multiple measures), Kruskal-Walllis test with Dunn’s multiple comparisons test (accounting for multiple measures), multiple t test with Holm-Sidak correction for multiple comparisons or Mann-Whitney tests, dependent upon normality of data.

## Supplementary information

Supplementary Information.

## Data Availability

The datasets generated during the current study are available from the corresponding author on reasonable request.

## References

[1] Parks DJ (1999). Bile acids: natural ligands for an orphan nuclear receptor. Sci..

[2] Sayin SI (2013). Gut microbiota regulates bile acid metabolism by reducing the levels of tauro-beta-muricholic acid, a naturally occurring FXR antagonist. Cell Metab..

[3] Maruyama T (2002). Identification of membrane-type receptor for bile acids (M-BAR). Biochem. Biophys. Res. Comm..

[4] Glantz A, Marschall HU, Mattsson LA (2004). Intrahepatic cholestasis of pregnancy: Relationships between bile acid levels and fetal complication rates. Hepatology.

[5] Ovadia C (2019). Association of adverse perinatal outcomes of intrahepatic cholestasis of pregnancy with biochemical markers: results of aggregate and individual patient data meta-analyses. Lancet.

[6] Martineau MG (2015). The metabolic profile of intrahepatic cholestasis of pregnancy is associated with impaired glucose tolerance, dyslipidemia, and increased fetal growth. Diabetes Care.

[7] Dann AT (2006). Plasma lipid profiles of women with intrahepatic cholestasis of pregnancy. Obstet. Gynecol..

[8] Kong X, Kong Y, Zhang F, Wang T, Yan J (2016). Evaluating the effectiveness and safety of ursodeoxycholic acid in treatment of intrahepatic cholestasis of pregnancy: A meta-analysis (a prisma-compliant study). Med..

[9] Geenes V (2014). The reversed feto-maternal bile acid gradient in intrahepatic cholestasis of pregnancy is corrected by ursodeoxycholic acid. PLoS One.

[10] Tsuchida T, Shiraishi M, Ohta T, Sakai K, Ishii S (2012). Ursodeoxycholic acid improves insulin sensitivity and hepatic steatosis by inducing the excretion of hepatic lipids in high-fat diet–fed KK-Ay mice. Metab..

[11] Koren O (2012). Host remodeling of the gut microbiome and metabolic changes during pregnancy. Cell.

[12] Gohir W (2015). Pregnancy-related changes in the maternal gut microbiota are dependent upon the mother’s periconceptional diet. Gut Microbes.

[13] Ovadia C (2019). Enhanced microbial bile acid deconjugation and impaired ileal uptake in pregnancy repress intestinal regulation of bile acid synthesis. Hepatology.

[14] Milona A (2010). Raised hepatic bile acid concentrations during pregnancy in mice are associated with reduced farnesoid X receptor function. Hepatology.

[15] Manna, L., *et al*. Enzymatic quantification of total serum bile acids as a monitoring strategy for women with intrahepatic cholestasis of pregnancy receiving ursodeoxycholic acid treatment: a cohort study. *BJOG* 10.111/1471-0528.15926 (2019). 10.1111/1471-0528.15926 PMC6899621 31483939

[16] Beuers U, Trauner M, Jansen P, Poupon R (2015). New paradigms in the treatment of hepatic cholestasis: from UDCA to FXR, PXR and beyond. J. Hepatol..

[17] Zhang Y (2017). Comparative potency of obeticholic acid and natural bile acids on FXR in hepatic and intestinal *in vitro* cell models. Pharmacol. Res. Perspect..

[18] Zhang JH (2013). Potent stimulation of fibroblast growth factor 19 expression in the human ileum by bile acids. Am. J. Physiol. Gastrointest. Liver Physiol.

[19] Hirano S, Masuda N, Oda H (1981). *In vitro* transformation of chenodeoxycholic acid and ursodeoxycholic acid by human intestinal flora, with particular reference to the mutual conversion between the two bile acids. J. Lipid Res..

[20] Crosignani A (1996). Clinical pharmacokinetics of therapeutic bile acids. Clin. Pharmacokinet..

[21] Brites D (1998). Correction of maternal serum bile acid profile during ursodeoxycholic acid therapy in cholestasis of pregnancy. J. Hepatol..

[22] Gadaleta RM (2018). Suppression of hepatic bile acid synthesis by a non-tumorigenic FGF19 analogue protects mice from fibrosis and hepatocarcinogenesis. Sci. Rep..

[23] Mueller M (2015). Ursodeoxycholic acid exerts farnesoid X receptor-antagonistic effects on bile acid and lipid metabolism in morbid obesity. J. Hepatol..

[24] Jiang C (2015). Intestine-selective farnesoid X receptor inhibition improves obesity-related metabolic dysfunction. Nat. Commun..

[25] Metsälä J, Stach-Lempinen B, Gissler M, Eriksson JG, Koivusalo S (2016). Risk of pregnancy complications in relation to maternal prepregnancy body mass index: population-based study from Finland 2006-10. Paediatr. Perinat. Epidemiol..

[26] Ley RE, Turnbaugh PJ, Klein S, Gordon JI (2006). Microbial ecology: Human gut microbes associated with obesity. Nat..

[27] Turnbaugh PJ (2009). A core gut microbiome in obese and lean twins. Nat..

[28] Armougom F, Henry M, Vialettes B, Raccah D, Raoult D (2009). Monitoring bacterial community of human gut microbiota reveals an increase in Lactobacillus in obese patients and Methanogens in anorexic patients. PLoS One.

[29] Zuo H-J (2011). Gut bacteria alteration in obese people and its relationship with gene polymorphism. World J. Gastroenterol..

[30] Mullish BH (2019). Microbial bile salt hydrolases mediate the efficacy of faecal microbiota transplant in the teratment of recurrent *Clostridioides difficile* infection. Gut.

[31] Aldini R (1996). Intestinal absorption of bile acids in the rabbit: different transport rates in jejunum and ileum. Gastroenterology.

[32] Enright EF (2018). Gut microbiota-mediated bile acid transformations alter the cellular response to multidrug resistant transporter substrates *in vitro*: focus on p-glycoprotein. Mol. Pharm..

[33] Sánchez-García A, Sahebkar A, Simental-Mendía M, Simental-Mendía LE (2018). Effect of ursodeoxycholic acid on glycemic markers: A systematic review and meta-analysis of clinical trials. Pharmacol. Res..

[34] Chappell LC (2019). Ursodeoxycholic acid versus placebo in women with intrahepatic cholestasis of pregnancy (PITCHES): a randomised controlled trial. Lancet.

[35] Brooks L (2017). Fermentable carbohydrate stimulates FFAR2-dependent colonic PYY cell expansion to increase satiety. Mol. Metab..

[36] RTL Genomics. Data Analysis Methodology for Microbial Diversity. https://static1.squarespace.com/static/5807c0ce579fb39e1dd6addd/t/5813af0fd482e97e5eb4fcb5/1477685010205/Data_Analysis_Methodology.pdf (2019).

[37] Mullish BH (2018). Functional microbiomics: evaluation of gut microbiota-bile acid metabolism interactions in health and disease. Methods.

[38] Kozich JJ, Westcott SL, Baxter NT, Highlander SK, Schloss PD (2013). Development of a dual-index sequencing strategy and curation pipeline for analyzing amplicon sequence data on the MiSeq Illumina sequencing platform. Appl. Env. Microbiol..

[39] Glöckner FO (2017). 25 years of serving the community with ribosomal RNA gene reference databases and tools. J. Biotechnol..

[40] Wang Q, Garrity GM, Tiedje JM, Cole JR (2007). Naive Bayesian classifier for rapid assignment of rRNA sequences into the new bacterial taxonomy. Appl. Env. Microbiol..

[41] Morris LS, Marchesi JR (2016). Assessing the impact of long term frozen storage of faecal samples on protein concentration and protease activity. J. Microbiol. Methods.

[42] Smith K, Zeng X, Lin J (2014). Discovery of bile salt hydrolase inhibitors using an efficient high-throughput screening system. PLoS One.

[43] Tremaroli V (2015). Roux-en-Y gastric bypass and vertical banded gastroplasty induce long-term changes on the human gut microbiome contributing to fat mass regulation. Cell Metab..

