# Supplementary material for: Ursodeoxycholic acid enriches intestinal bile salt hydrolase-expressing Bacteroidetes in cholestatic pregnancy
Source: Sci Rep. 2020 Mar 3;10:3895. doi: 10.1038/s41598-020-60821-w (PMC7054423; doi:10.1038/s41598-020-60821-w)
Supplement: Supplementary file 1 — Supplementary Information. [file 41598_2020_60821_MOESM1_ESM.pdf]

Supplementary Information

Ursodeoxycholic acid enriches intestinal bile salt hydrolase-expressing *Bacteroidetes* in cholestatic pregnancy

Caroline Ovadia, Alvaro Perdones-Montero, Hei Man Fan, Benjamin H. Mullish, Julie A.K. McDonald, Georgia Papacleovoulou, Annika Wahlström, Marcus Ståhlman, Anastasia Tsakmaki, Louise C.D. Clarke, Alexandros Sklavounos, Peter H. Dixon, Gavin A. Bewick, Julian R.F. Walters, Hanns-Ulrich Marschall, Julian R. Marchesi, Catherine Williamson

Contents

Supplementary Figure S1 ....Page 2
Supplementary Figure S2 ....Page 3
Supplementary Figure S3 ....Page 4
Supplementary Figure S4.. Page 5
Supplementary Figure S5 ....Page 6
Supplementary Table S1 .... Page 7
Supplementary Table S2 .... Page 7
Supplementary Table S3.. Page 8

Supplementary Figure S1

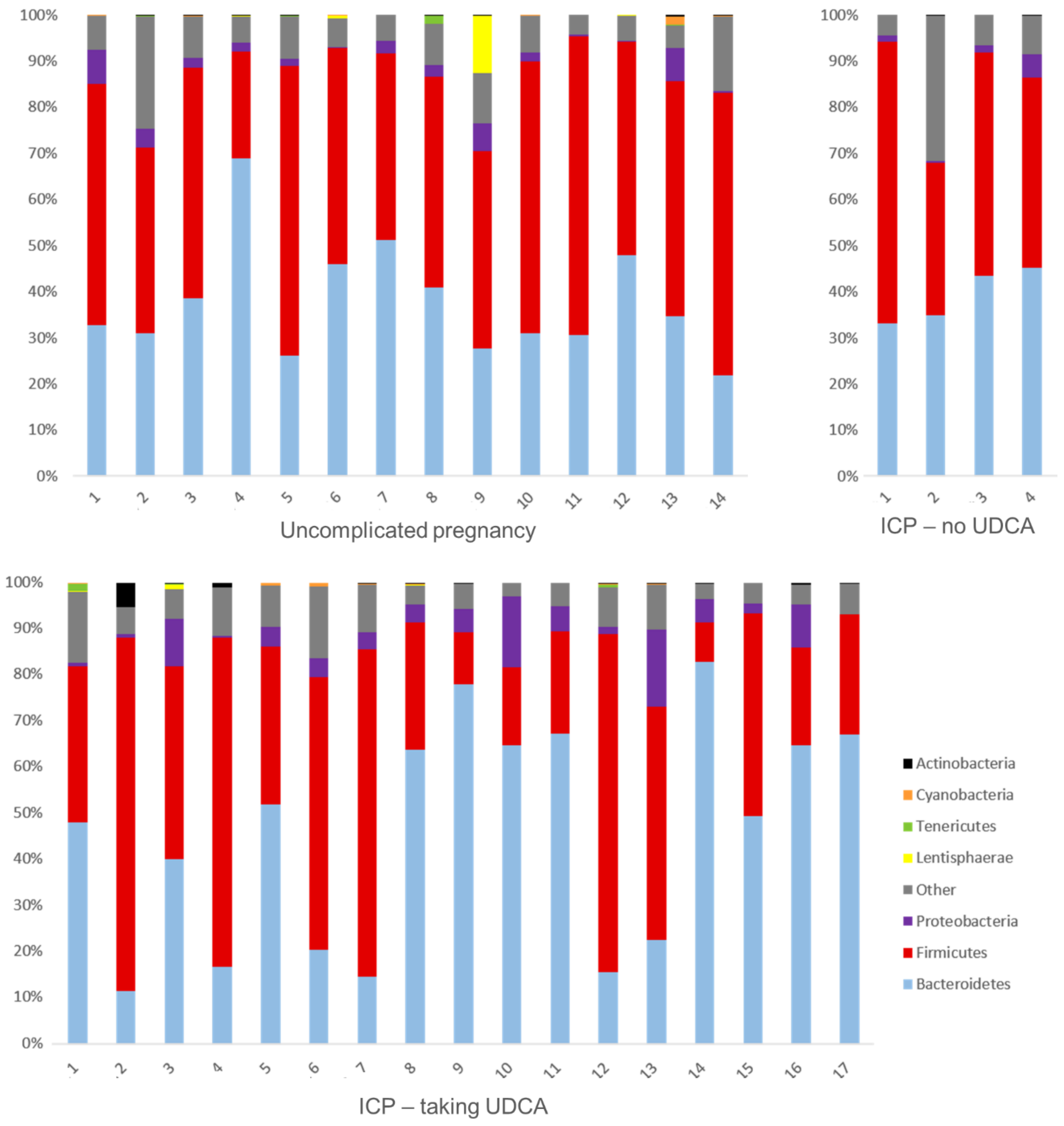

Individual distribution of microbes at phylum level in women with normal pregnancies (n=14), women with intrahepatic cholestasis of pregnancy (ICP) treated with ursodeoxycholic acid (UDCA, n=17), and women with untreated ICP (n=4).

Supplementary Figure S2. The faecal microbiota of cholestatic and uncomplicated pregnancy cluster according to order level according to the ratio of *Bacteroidetes* to *Firmicutes*

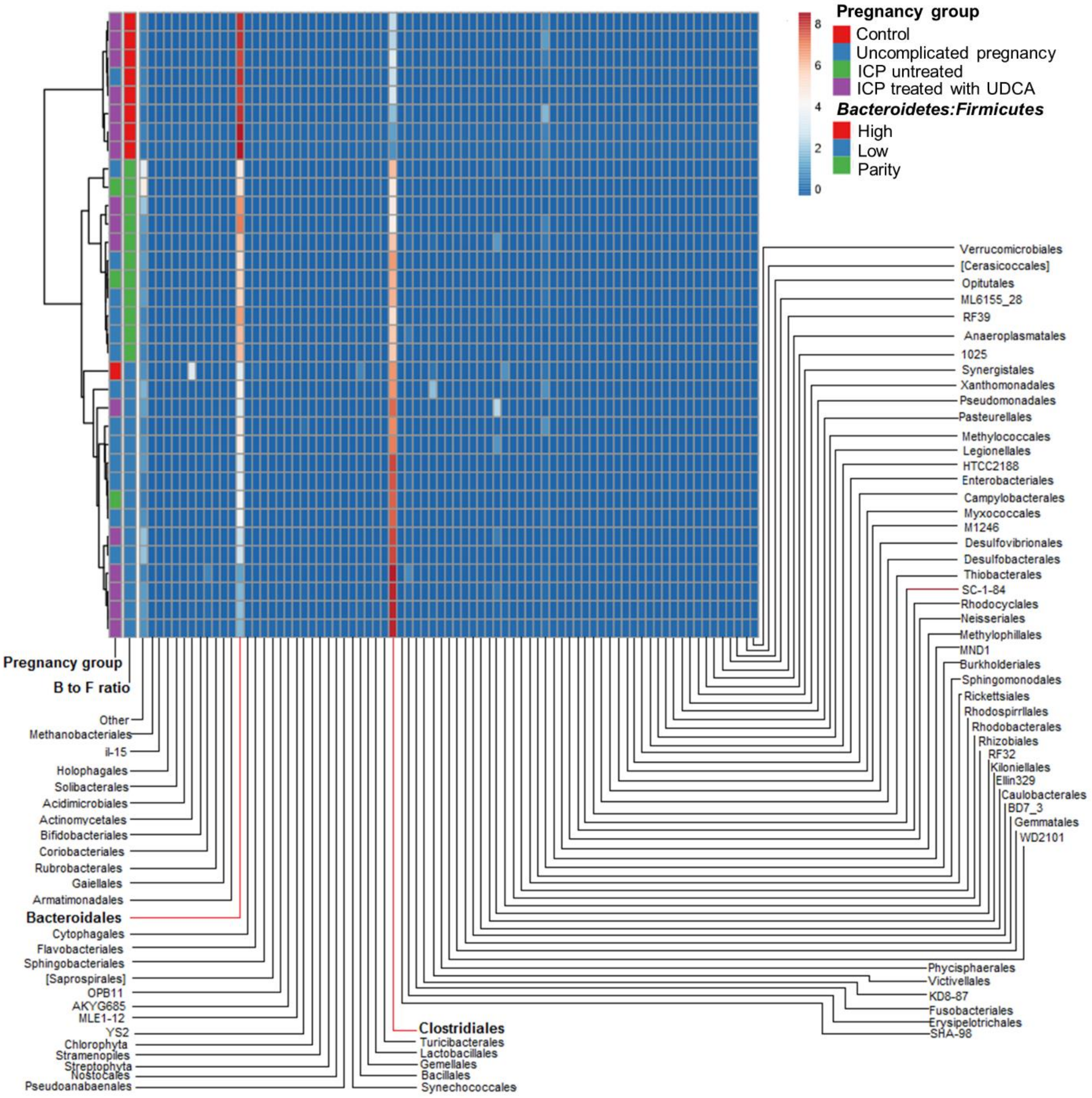

Heat map demonstrating unsupervised clustering of faecal samples by microbiota profiles determined from 16S rRNA gene sequencing, according to *B*:*F* (the ratio of *Bacteroidetes* to *Firmicutes*), at order level. Each row refers to faecal samples from women with normal pregnancies (n=14), women with intrahepatic cholestasis of pregnancy (ICP) treated with ursodeoxycholic acid (UDCA, n=17), and women with untreated ICP (n=4). Uncomplicated pregnancy: blue status; ICP treated with UDCA: purple status; untreated ICP: green status; red status shows methodological control. Box colours show relative bacterial abundance, dark blue reflecting low proportion of sequences present in samples and red showing high proportions sequences present in samples.

Supplementary Figure S3. The effect of UDCA treatment in ICP on the serum ratio of cholic acid to chenodeoxycholic acid

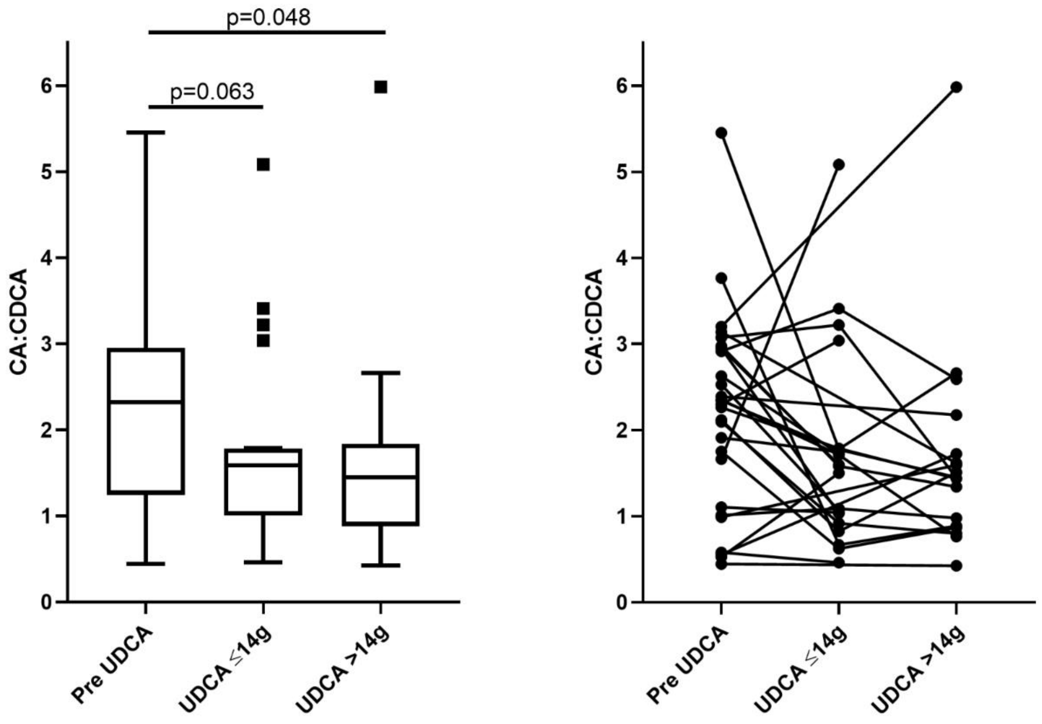

Ratio of serum cholic acid (CA) to chenodeoxycholic acid (CDCA) in 28 women with ICP treated with ursodeoxycholic acid (UDCA). Mean values taken from samples obtained following diagnosis of ICP and prior to commencing UDCA (Pre UDCA), having commenced UDCA treatment but in total receiving less than or equal to 14g UDCA (UDCA≤14g), or having received >14g UDCA (UDCA >14g) assuming compliance with prescribed medication. Groups were compared with Wilcoxon matched-pairs signed rank test and Bonferroni correction for multiple comparisons. Boxes show median and interquartile range (IQR), with whiskers at 1.5 IQR

Supplementary Figure S4

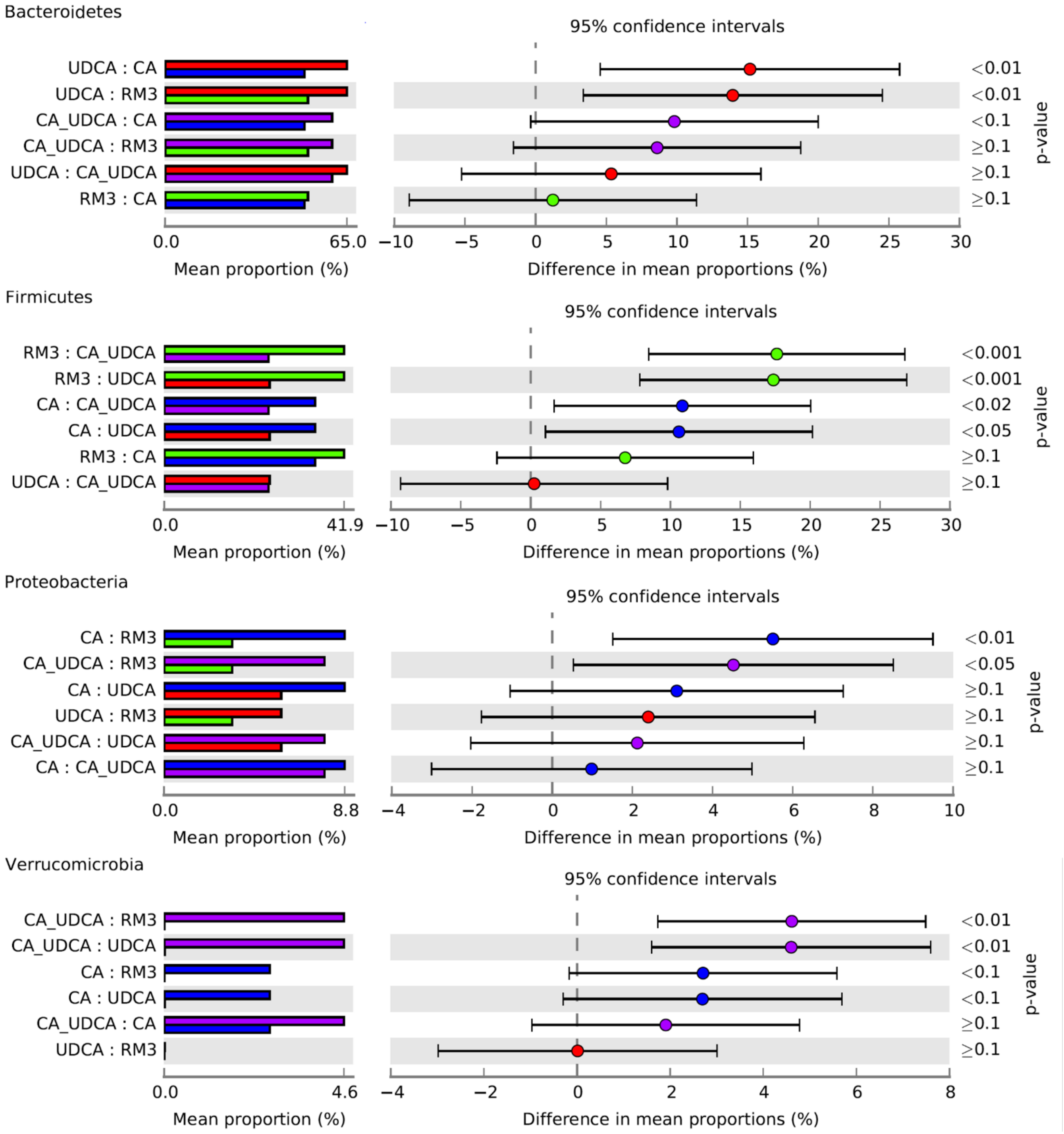

Comparison of proportions of main bacterial phyla between diet groups in mice with normal chow (RM3) diet, or supplemented with 0.5% cholic acid (CA), 0.5% ursodeoxycholic acid (UDCA), or 0.5% CA plus 0.5% UDCA (CA_UDCA). N=6-7 per group.

Supplementary Figure S5. Richness and diversity of caecal microbiota for pregnant mice according to diet.

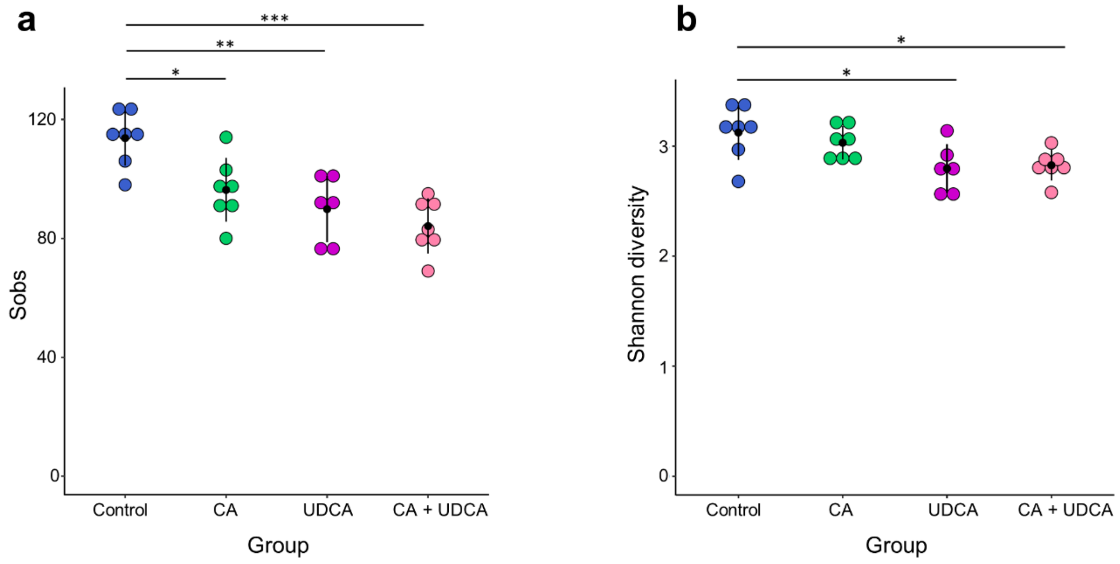

Individual results and mean for **(a)** richness and **(b)** diversity of caecal microbiota for mice fed a normal chow diet (n=7) (control), or one supplemented with 0.5% cholic acid (n=7) (CA), 0.5% ursodeoxycholic acid (UDCA) (n=6), or 0.5% CA and 0.5% UDCA (n=7). Sobs: total number of species observed in a sample.

Supplementary Table S1. Clinical features of patient groups

	ICP – on UDCA	ICP – no UDCA		Control pregnant	
			Comparison with UDCA		Comparison with UDCA
**Number of women**	17	4	-	14	-
**Maternal age (years)**	36 (30 to 38)	32 (28 to 36)	ns	34 (32 to 36)	Ns
**Gestation of sample (week^{+day})**	36^{+4} (33^{+0} to 37^{+0})	36^{+2} (35^{+2} to 38^{+5})	ns	35^{+4} (34^{+2} to 39^{+3})	Ns
**Peak bile acid concentration pre-sample (µmol/L)**	48 (36 to 89)	19 (13 to 33)	p=0.0337	4 (2 to 5)	p<0.0001
**Bile acid concentration at time of sample (µmol/L)**	34 (21 to 64)	18 (8 to 32)	ns	4 (3 to 6)	p=0.0060
**Peak bile acid concentration throughout pregnancy (µmol/L)**	75 (48 to 139)	32 (20 to 49)	p=0.0059	4 (3 to 6)	P<0.0001

ICP: intrahepatic cholestasis of pregnancy, UDCA: ursodeoxycholic acid, ns: not significant. Groups compared with 2 way ANOVA, p<0.05 used as threshold for significance.

Supplementary Table S2. Effect of UDCA treatment on the ratio of serum CA:CDCA

Sample comparison	CA:CDCA increase % (n)	CA:CDCA decrease % (n)
**Pre UDCA → ≤14g UDCA**	26% (5)	74% (14)
**Pre UDCA → >14g UDCA**	17% (3)	83% (15)
**≤14g UDCA → >14g UDCA**	33% (4)	67% (8)

Proportion (%) and number of women with ICP for whom mean serum cholic acid (CA) to chenodeoxycholic acid (CDCA) ratio changed following initiation of UDCA treatment, by total dose of UDCA taken before serum sample was collected.

Supplementary Table S3. Comparative caecal microbiota between dietary groups

Diet group comparison	*F* statistic	R^2	Adjusted p value
**Control vs CA**	9.48	0.44	0.006
**Control vs UDCA**	5.06	0.31	0.014
**Control vs CA + UDCA**	17.33	0.59	0.006
**CA vs UDCA**	3.45	0.24	0.042
**CA vs CA + UDCA**	2.18	0.15	0.088
**UDCA vs CA + UDCA**	2.32	0.17	0.066

Comparisons between groups were performed using PERMANOVA in R. N=6-7 per group.
